# Hospital mortality of adults admitted to Intensive Care Units in hospitals with and without Intermediate Care Units: a multicentre European cohort study

**DOI:** 10.1186/s13054-014-0551-8

**Published:** 2014-10-09

**Authors:** Maurizia Capuzzo, Carlo Alberto Volta, Tania Tassinati, Rui Paulo Moreno, Andreas Valentin, Bertrand Guidet, Gaetano Iapichino, Claude Martin, Thomas Perneger, Christophe Combescure, Antoine Poncet, Andrew Rhodes, on behalf of the Working Group on Health Economics of the European Society of Intensive Care Medicine

**Affiliations:** Section of Anaesthesia and Intensive Care, Department of Morphology, Surgery and Experimental Medicine, S. Anna Hospital, University of Ferrara, Via Aldo Moro 8, 44124 Cona, Ferrara, Italy; Unidade de Cuidados Intensivos Neurocríticos, Hospital de São José, Centro Hospitalar de Lisboa Central, Rua José António Serrano Lisboa, 1150-199 Portugal; Rudolfstiftung Hospital and Medical University of Vienna, General and Medical Intensive Care Unit (Rudolfstiftung Hospital), Juchgasse 25, 1030 Vienna, Austria; Assistance Publique - Hôpitaux de Paris, Hôpital Saint-Antoine, Service de Réanimation Médicale, 184 rue du Faubourg Saint-Antoine, Paris, F-75012 France; UPMC Univ Paris 06; Inserm, Unité de Recherche en Épidémiologie Systèmes d’Information et Modélisation (U707), 4 Place Jussieu, Paris, F-75012 France; Dipartimento di Fisiopatologia Medico-Chirurgica e dei Trapianti, Università degli Studi di Milano, Polo Universitario San Paolo, Via Ovada 26, 20142 Milan, Italy; Department of Anaesthesia and Intensive Care, Hôpital Nord, Chemin des Bourrely, F-13015 Marseille, France; Division of Clinical Epidemiology, University Hospitals of Geneva and University of Geneva, 24 Micheli-du-Crest, CH-1211 Geneva, Switzerland; Department of Intensive Care Medicine, St George’s Healthcare NHS Trust and University of London, Blackshaw Road, London, SW17 0QT UK

## Abstract

**Introduction:**

The aim of the study was to assess whether adults admitted to hospitals with both Intensive Care Units (ICU) and Intermediate Care Units (IMCU) have lower in-hospital mortality than those admitted to ICUs without an IMCU.

**Methods:**

An observational multinational cohort study performed on patients admitted to participating ICUs during a four-week period. IMCU was defined as any physically and administratively independent unit open 24 hours a day, seven days a week providing a level of care lower than an ICU but higher than a ward. Characteristics of hospitals, ICUs and patients admitted to study ICUs were recorded. The main outcome was all-cause in-hospital mortality until hospital discharge (censored at 90 days).

**Results:**

One hundred and sixty-seven ICUs from 17 European countries enrolled 5,834 patients. Overall, 1,113 (19.1%) patients died in the ICU and 1,397 died in hospital, with a total of 1,397 (23.9%) deaths. The illness severity was higher for patients in ICUs with an IMCU (median Simplified Acute Physiology Score (SAPS) II: 37) than for patients in ICUs without an IMCU (median SAPS II: 29, *P* <0.001). After adjustment for patient characteristics at admission such as illness severity, and ICU and hospital characteristics, the odds ratio of mortality was 0.63 (95% CI 0.45 to 0.88, *P* = 0.007) in favour of the presence of IMCU. The protective effect of the IMCU was absent in patients who were admitted for basic observation, for example, after surgery (odds ratio 1.15, 95% CI 0.65 to 2.03, *P* = 0.630) but was strong in patients admitted to an ICU for other reasons (odds ratio 0.54, 95% CI 0.37 to 0.80, *P* = 0.002).

**Conclusions:**

The presence of an IMCU in the hospital is associated with significantly reduced adjusted hospital mortality for adults admitted to the ICU. This effect is relevant for the patients requiring full intensive treatment.

**Trial registration:**

Clinicaltrials.gov NCT01422070. Registered 19 August 2011.

**Electronic supplementary material:**

The online version of this article (doi:10.1186/s13054-014-0551-8) contains supplementary material, which is available to authorized users.

## Introduction

The Intensive Care Unit (ICU) is the part of the hospital where care is provided to the sickest patients. It is typified by having a high level of monitoring and therapeutic technologies, a very high degree of organization and high staff to patient ratios. Despite the high severity of illness of patents admitted to ICU, most improve to the point to be discharged to a normal ward care environment. A significant proportion of these ICU-discharged patients subsequently die in the hospital with post-ICU mortality rates ranging from 6 to 27% [1-7] either as a result of residual organ dysfunction/failure or due to the inability of the staff in lower levels of care to cope appropriately with the needs of these patients [8].

Premature discharge from ICU is more likely to occur at night due to the pressure for beds on ICU, and is associated with higher risk of death [9]. Suggested factors that might account for a worse outcome of prematurely discharged patients are inferior quantities and qualities of care available both during the transfer and at the destination. To facilitate earlier ICU discharge for patients needing more care than could be provided on wards, Intermediate Care Units (IMCUs), with a level of nursing staff (and costs) lower than ICU although higher than the general wards, have been proposed [10-13]. Other positive effects of the presence of an IMCU include a reduction in the number of unplanned readmissions to ICU as a consequence of providing more monitoring and nursing care than is available on hospital wards [14-16] and a decrease in hospital mortality rates due to a lower pressure on the availability of beds in ICUs [17]. Moreover, an IMCU may also act as a step-up unit for patients deteriorating on wards ensuring timely care, and specialized IMCUs like coronary, respiratory or stroke units can treat patients never needing intensive care admission. This later effect is highly debated, since it can delay the immediate admission of a patient with impending critical illness to the ICU, just wasting time for the patient to receive the appropriate level of care.

The efficacy of IMCUs in Europe has been questioned [18] and the pertinent literature shows variable results. In a study performed on the EURICUS-I database [19] the sensitivity analysis on in-hospital mortality showed that patients discharged to IMCUs had a better outcome than patients discharged to the ward. Beck *et al*. [20] found a higher risk of post-ICU mortality for late (20.00 h to 07.59 h) discharges to hospital wards in comparison with late discharges to IMCU. More recently, an evaluation of the modernisation of adult critical care services in England showed that the increase in the number of staffed ICU beds started by the Department of Health in 2000 involved more high dependency than intensive care beds (increased by 106% and 23%, respectively), and was associated with reductions in the adjusted mortality, and both transfers between units and unplanned night discharges [21]. On the other hand, a study comparing patients admitted to IMCU with low-risk ICU patients [22] reported that the former had significantly higher hospital mortality than the latter, despite a lower severity of illness; however, there were differences in the IMCU and ICU case mix. More recently, Peelen *et al*. [23] who studied severe sepsis patients admitted to Dutch ICUs found that the presence of an IMCU as a step-down facility was associated with greater in-hospital mortality. Among the possible explanations, the authors mention hospital case-mix differences, unrevealed confounders but also the possibility of premature discharge when an IMCU is available. Moreover, Solberg *et al*. did not find a decrease in ICU readmissions after introducing an IMCU [24] while Keegan *et al*. found an increase of ICU readmission after the introduction of a non-intensivist-directed speciality-specific progressive care unit [25]. Overall, the potential effect of an IMCU can be assigned to a higher nurse to patient ratio than the one existing in regular wards [26] and/or its ability to cope with residual patient organ dysfunction/failures [8].

The primary aim of this observational multinational European cohort study was to assess whether the patients admitted to ICUs with an IMCU in the hospital have lower hospital mortality than those admitted to ICUs without an IMCU in the same hospital.

## Material and methods

The European Mortality and Length Of ICU Stay (ELOISE) study was designed and endorsed by the Working Group on Health Economics of the European Society of Intensive Care Medicine (ESICM). The country coordinators (listed in the Appendix) directly approached colleagues to invite them to participate and helped them obtain any regulatory authority approvals as appropriate. Local study coordinators (listed in the Appendix) were responsible for obtaining any applicable permissions from local ethics bodies, answering the study unit questionnaire, training their colleagues and supervising the daily collection of patient data, getting hospital discharge data, transmitting patient data without any personally identifiable information to the Coordination and Communications Centre (CCC), and performing data re-abstraction of selected cases for quality control. During the study period, the CCC was active for management of the website [27], assignment of code to each study unit, dissemination of information, help in solving problems concerning definitions and software, and periodic email transmission of reminders.

The ethics requirements in different countries and the list of the ethics bodies that approved the study are reported in the acknowledgements section.

### Study unit questionnaire

This questionnaire was discussed in the Working Group of ESICM and finalized by the members of the Steering Committee (listed in the Appendix). It was designed to collect information about the unit and the hospital where the unit was located. However, we did not formally validate our study unit questionnaire. Each local coordinator answered the questionnaire and reported the highest Level of Care (LOC) provided by the participating unit to the patients. The LOC was defined according to the recently published ESICM recommendations on basic requirements for ICUs [28] where LOC III represents patients with multiple acute vital organ failure, LOC II represents patients requiring monitoring and pharmacological and/or device-related support of only one acutely failing vital organ system, and LOC I patients experience signs of organ dysfunction necessitating continuous monitoring and minor pharmacological or device-related support. For the present study an IMCU was defined as any physically and administratively independent unit providing LOC I/II to patients open twenty-four hours per day, seven days per week.

Local coordinators collected data on the hospital characteristics (number of acute care beds and annual number of hospital admissions), and numbers of LOC III, II and I units present in the hospital. They provided information about the organization of the study unit including the number of active beds and actual staffing. Some ICUs reported having intermediate care beds physically included in the unit. Therefore, to analyse nurse to patient ratios of these ICUs the number of ICU beds was adjusted considering that two intermediate care beds inside the ICU equal one ICU bed [28]. The local coordinators were also asked as to whether there was any possibility of allocating extra beds inside the unit when necessary.

### Data collection

An Excel file with plausibility limits was provided to participating units by the CCC through the website, where the study protocol, Case Report Form and detailed definitions of the variables were available. All patients aged ≥16 years, consecutively admitted to a participating unit during the study period, not admitted only for organ donation, and without any limitations of care at ICU admission were included. Informed consent was waived for the ICUs of some countries (Austria, Czech Republic, Denmark, Germany, France, Norway, Poland), while in other countries it was required by some ethics bodies but not by others. Accordingly, the local study coordinators obtained the patient consent to participate in the study where appropriate. Participating units chose one of two available study periods (either from 7 November to 4 December 2011, or from 16 January to 12 February 2012) for patient data collection. The maximum number of admissions collected by each unit was limited to 100.

The patient data collected for the study included variables to compute Simplified Acute Physiology Score (SAPS) II [29] and SAPS 3 at admission [6,30], and Sequential Organ Failure Assessment (SOFA) [31] and nursing workload index (NEMS) [32] on the last day in the study unit for survivors. A follow-up until hospital discharge was performed and censored at 90 days after admission to the study unit, and date, time, vital status at hospital discharge as well as any transfer to a LOC higher than ward after discharge from the study unit and before hospital discharge were recorded. When a patient was discharged from the study unit to another acute hospital, date, time and vital status at hospital discharge were assumed to be the same as unit discharge. For the calculation of each severity score, if the number of missing values for a single admission was ≤3 the missing values were scored as normal. When more than three values were missing, the entire score was considered as missing. All the lengths of stay were computed using exact days (number of hours/24) but for cases missing any information on time, we calculated lengths of stay according to the rule proposed by Ruttiman and Pollack [33].

At the end of the study period, each study unit was required to re-abstract the data of a maximum of three cases identified by the CCC for quality control.

### Statistical analysis

Quality control assessment was performed comparing data of re-scored patients to their original counterparts through kappa coefficients and intraclass correlation coefficients, as appropriate.

Categorical variables are described as counts and percentages, and continuous variables as mean and standard deviation if normally distributed, or median with interquartile (IQR) range. Comparisons between patients in units with and without an IMCU in the hospital were performed using chi-squared or Fisher exact test, and Student *t* test.

Regression analyses were conducted to assess the association between the availability of IMCU and hospital mortality. As the availability of an IMCU is a centre-level factor, generalized estimating equation (GEE) models were used to account for the correlation of patients within centres [34]. GEE produces estimates comparable to those from ordinary logistic regression but adjusts the confidence interval for the correlation of outcomes within-centre.

Univariate odds ratios (ORs) were reported with 95% confidence intervals (CI). The log-linearity of the SAPS II parameter was checked. A multivariable analysis was conducted to adjust for the potential confounders selected *a priori* by the authors. They included gender and patient level factors related to health status at admission (‘basic observation’ as reason for ICU admission, SAPS II, infection, planned/unplanned admission to the ICU, number of days in hospital before ICU admission and intra-hospital location before ICU admission), characteristics of units or hospitals (number of hospital beds, adjusted number of ICU beds) and countries. The organization of ICU was captured by the following factors: possibility of allocating extra beds inside the ICU, having intermediate care beds inside the ICU and ICU nurse to patient ratio during daytime hours. A model with an interaction term was also performed to test the modification of the effect of presence of an IMCU according to the reason of admission (‘basic observation’ versus reasons requiring intensive treatment).

### Ethical approval

Ethics requirements differed by country. Given the design of ELOISE study, and given the regulations in Austria, Poland and Switzerland no ethics approval was required. In France, the ‘Groupe Ethique de l’association pour la Formation et la Recherché en anesthésie-réanimation’ approved the study. In the UK, the National Research Ethics Committee London - Harrow approved the study. In some countries (Belgium, Denmark, and Norway), the ethical approval obtained by the coordinating centre was valid for all the centres in the same country. In some countries (Ireland, Italy), ethics requirements differed by centres of the same country. Moreover, in some centres, the study was considered and managed as an audit. However, each unit was responsible for obtaining local permissions, as necessary, according to local regulations.

The following ethical bodies approved the study: Commissie voor Medische Ethiek - Ghent University Hospital; Comité d’éthique des Cliniques de l’Europe; Comité d’ethique Hospitalo-Facultaire Universitaire de Liège; Ethisch Comité Onze Lieve Vrouwziekenhuis Aalst; Ethics Committee of the Teaching Hospital and Medical Faculty Plzen; Etická komise FN Brno; Ethics Committee of the University Hospital in Hradec Kralove; Regional Scientific Ethics Committee of Southern Denmark; Ethics Committee of the University of Leipzig, Germany; Ethik-Kommission der Medizinischen Fakultät der Ruhr Universität Bochum, Germany; Scientific Committee of Attikon University Hospital; Scientific Board of G. Gennimatas General Hospital, Thessaloniki; Scientific Board of AHEPA University General Hospital of Thessaloniki; Scientific Committee of Aretaieion University Hospital, Athens; Ethics Committee of the University Hospital of Larissa; University Hospital of Ioannina Ethics Committee; Scientific Council of Hippokration General Hospital of Thessaloniki; Sotiria Hospital Ethics Committee, Athens; Ethics Committee of Papanikolaou Hospital, Thessaloniki; Scientific Committee of ‘Agioi Anargyroi’ Hospital, Athens; Naval Hospital of Athens Ethics Committee; Scientific Board of Sismanoglio General Hospital; Scientific Committee of IASO Center Thessalias; Scientific Committee of Artas General Hospital; Clinical Research Ethics Committee of the Cork Teaching Hospitals; Ethics (Medical Research) Committee, Beaumont Hospital, Dublin; Ethics and Medical Research Committee, St Vincent’s Healthcare Group Ltd.; Comitato Etico Indipendente dell’Azienda Ospedaliero-Universitaria di Bologna; Comitato Etico della Provincia di Ferrara; Comitato Etico Interaziendale AUSL Bologna e Imola; Comitato bioetico dell’ARNAS Ospedale Civico Di Cristina Benfratelli di Palermo; Modena Local Ethics Committee; Comitato Etico Azienda Ospedaliera San Paolo, Milano; Medical and Health Research Ethics Committee of REK Sør-Øst. Centre: Stavanger University Hospital; REK Sør-Øst. Centre: Ålesund Hospital; Comissão de Ética para a Saúde do CHLC; Comissão de Ética para a Saúde do Centro Hospitalar de Coimbra; Unidade Local de Saúde de Matosinhos Ethics Committee; Comissão de Ética da Unidade Local de Saúde do Alto Minho; Comissão de Ética para a Saúde do Hospital S. João; Comissão de Ética para a Saúde do Centro Hospitalar de Setúbal; Ethics Committee of Emergency County Hospital Cluj-Napoca; Ethics Committee of Emergency Institute of Cardiovascular Diseases ‘Prof. Dr. C. C. Iliescu’, Bucharest, Romania; University Emergency County Hospital Mures Local Ethics Committee; Comisia Locala de Etica - Spitalul Universitar de Urgenta Elias; Ethics Committee of Emergency Institute of Cardiovascular Diseases “Prof. Dr. C. C. Iliescu”, Bucharest, Romania; Clinical Emergency Hospital of Bucharest Local Ethics Committee; Ethics Committee of Clinical Emergency County Hospital Timisoara; Education and Medical Research Committee of Spitalul Judetean de Urgenta ‘Dr. Constantin Opris’ Baia Mare; Consiliul Etical Institutul Clinic Fundeni Center; Comité Ético de Investigación Clínica de Cartagena; Investigation Committee of Hospital Universitario de Torrejón; Comité de Etica de Investigación Clínica de la Universidad de Navarra; Istanbul University Cerrahpasa Medical School, Clinical Research Ethics Committee; Ethics Committee of the Ankara Numune Training and Research Hospital; Clinical Research Ethics Committee of Tepecik Training and Research Hospital; Mersin University Clinical Research Ethics Committee; Bakırköy Dr. Sadi Konuk Education and Research Hospital.

## Results

We collected data for 6,401 admissions to 169 participating units in 17 European countries. Data quality control was performed on 281 (4%) records. The median number of missing data was 0.29 (IQR 0.11 to 0.62) per unit. Data quality was excellent (Additional file 1), as most reliability coefficients exceeded 0.85. Only ‘transfer to higher LOC before ICU’ and ‘Readmission’ had borderline kappa values (0.842 and 0.838, respectively).

Of the participating units, 167 (98.8%) qualified themselves as being able to provide LOC III, which is to care for patients with multiple acute vital organ failure who cannot be accommodated in other units. The remaining two units (from Austria and France) qualified themselves as only able to provide LOC I and II, respectively. To make the study sample as homogeneous as possible, the subsequent analysis was done on the data collected from the 167 units providing LOC III as the highest LOC, and they will be named ICUs hereafter.

Most of the ICUs (140 of 167, 84%) were in a hospital with at least one independent IMCU. This proportion ranged from 70% (Greece) to 100% (Portugal) in the countries represented by more than eight ICUs (Additional file 2). Only 31 of these ICUs (22.1%) were in hospitals with only one IMCU. The median number of IMCUs present in the hospitals was three (IQR 2 to 4.25). The most represented specialities of IMCUs were cardiology (present in 93), surgery (62) including general and speciality, internal medicine (38), neurology (38), and emergency (17), while 23 IMCU were mixed. The median number of IMCU beds in the hospital was 12 (IQR 4 to 20) for an IMCU providing LOC II (monitoring and pharmacological and/or device-related support of only one acutely failing vital organ system) and 10 (IQR 4 to 24) for those providing LOC I (monitoring and minor pharmacological or device-related support).

The number of acute hospital beds and the number of ICU staffed beds, both absolute and adjusted, were significantly higher in ICUs with an IMCU in the hospital than in those without it (organisational characteristics of study ICUs in Additional file 3). Fifty-one of the ICUs in hospitals with an IMCU (36.4%) and seven (25.9%) of the ICUs in hospitals without an IMCU had some intermediate care beds inside the ICU.

**Figure 1:**
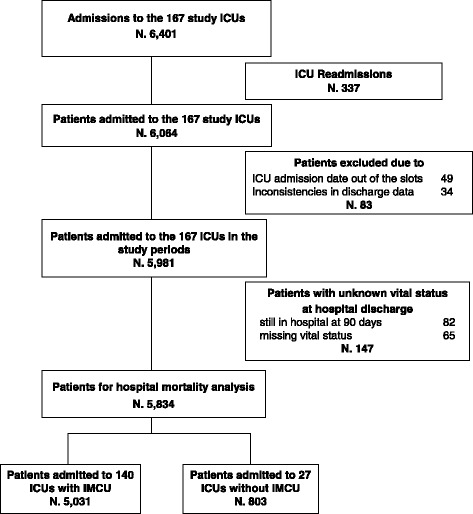
**Flowchart of the patients included in the study.**

There were 6,401 admissions collected by the study ICUs (Figure 1), 2,625 collected by 64 ICUs in the first, and 3,776 by 103 ICUs in the second slot period. The median number of admissions collected by each ICU was 32 (IQR 20 to 53). The exclusion of re-admissions during the same hospital course (337), of cases with ICU admission date out of the slots (49), or inconsistencies in discharge data (34), or unknown vital status at hospital discharge (82 still in hospital at 90-day follow-up, and 65 missing) left 5,834 patients for the analysis. Of the 5,834 patients studied, 1,397 (23.9%) died in hospital of which 1,113 (19.1%) died in ICU. The numbers of patients admitted to ICUs with and without an IMCU in the hospital were 5,031 (86.2%) and 803 (13.8%), respectively.

**Figure 2:**
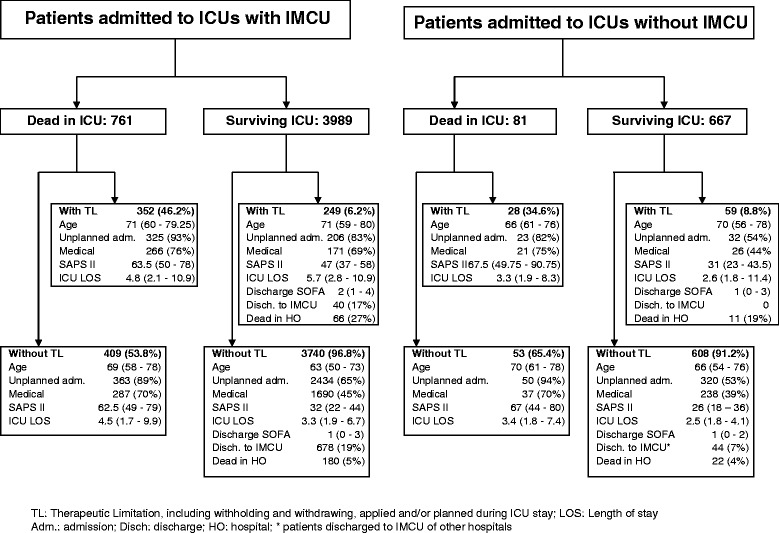
**Therapeutic limitation, including withholding and withdrawing, applied and/or planned during intensive care unit (ICU) stay.** Data on 4,750 (94.4%) patients admitted to ICUs with an Intermediate Care Unit (IMCU) and 748 (93.1%) patients admitted to ICUs without IMCU.

The patient and hospital characteristics according to the admission to ICU with or without an IMCU in the hospital are described in Table 1 and the reasons for ICU admission are reported in Additional file 4. The illness severity (especially SAPS II) was higher and ICU admissions were more frequently unplanned for patients in ICUs with an IMCU than for patients in ICUs without an IMCU. In agreement with the observed severity of illness of patients, crude hospital mortality was higher in ICUs with an IMCU (1232/5031, 24.5%) than in ICUs without an IMCU (165/803, 20.5%, *P* = 0.017). The IMCU was the discharge location for 721 (18.8%) of the 4,049 survivors of ICUs with an IMCU in the hospital while 44 (6.7%) of the 572 survivors of ICUs without an IMCU were discharged to an IMCU of another hospital. Information about therapeutic limitations was missing in 336 cases. In the 5,498 patients (94.2%) having information, recorded therapeutic limitations were applied during ICU stay and/or planned at ICU discharge in 601 (12.6%) and 87 (11.6%) patients admitted respectively to ICUs with and without an IMCU. Main characteristics of patients with and without any therapeutic limitation are reported in Figure 2. The SOFA score at ICU discharge was not significantly different in patients discharged from ICUs with and without an IMCU in the hospital (median (IQR): 1 (0 to 3) versus 1 (0 to 2), *P* = 0.361). NEMS at admission was higher in patients in ICUs with an IMCU (median (IQR): 29 (23 to 38) versus 27 (18 to 34), *P* <0.001) whereas NEMS at ICU discharge was similar (median (IQR) 18 (15 to 20) versus 18 (15 to 18), *P* = 0.89). Furthermore, the length of stay in an ICU with an IMCU was longer than in ICU without an IMCU (median (IQR) 3.5 (1.9 to 6.9) versus 2.6 (1.8 to 4.3), *P* <0.001). These findings suggest that the discharge policy is not different between the ICUs with an IMCU and ICUs without an IMCU, the patients are discharged at equivalent NEMS.

**Table 1:** **Patient and hospital characteristics according to the absence or presence of an Intermediate Care Unit in the hospital**

**Patients admitted to ICU**		**With IMCU**	**Without IMCU**	***P*** **value**
Number		5031	803	
Gender	Female	2002 (39.8%)	332 (41.3%)	0.427
	Male	3029 (60.2%)	471 (58.7%)	
Age, years	Median (IQR)	65 (52-75)	68 (55-77)	<0.001
	Missing	14	2	
Transfer to higher LOC before ICU admission	No	4464 (88.9%)	785 (97.8%)	<0.001
	Yes	558 (11.1%)	18 (2.2%)^a^	
	Missing	9	0	
Intra-hospital location before ICU admission	Emergency room	1828 (36.7%)	218 (27.2%)	<0.001
	Intermediate care	338 (6.8%)	33 (4.1%)^a^	
	Other ICU	243 (4.9%)	27 (3.4%)	
	Ward, other	2571 (51.6%)	523 (65.3%)	
	Missing	51	2	
ICU admission	Planned	1471 (29.3%)	331 (41.2%)	<0.001
	Unplanned	3554 (70.7%)	472 (58.8%)	
	Missing	6	0	
‘Basic observation’ as ICU admission reason	No	3804 (75.6%)	475 (59.2%)	<0.001
	Yes	1227 (24.4%)	328 (40.8%)	
Surgery	Emergency surgery	993 (19.8%)	128 (15.9%)	<0.001
	No surgery	2603 (51.8%)	361 (45%)	
	Scheduled surgery	1426 (28.4%)	314 (39.1%)	
	Missing	9	0	
Infection at ICU admission	No	3461 (69.9%)	652 (81.5%)	<0.001
	Yes	1492 (30.1%)	148 (18.5%)	
	Missing	78	3	
SAPS II	Median (IQR)	37 (24-53)	29 (20-45)	<0.001
SAPS II predicted mortality	Median (IQR)	0.20 (0.06-0.53)	0.10 (0.04-0.35)	<0.001
	Missing	59	3	
SAPS 3	Median (IQR)	35 (23-48)	28 (19-41)	<0.001
SAPS 3 predicted mortality	Median (IQR)	0.19 (0.06-0.44)	0.10 (0.04-0.30)	<0.001
	Missing	55	7	
ICU length of stay, days	Median (IQR)	3.7 (1.9-7.7)	2.8 (1.8-4.8)	<0.001
	Missing	48	5	
ICU outcome	Survival	4049 (80.5%)	672 (83.7%)	0.036
	Death	982 (19.5%)	131 (16.3%)	
Hospital length of stay, days	Median (IQR)	13.9 (7.6-25)	11.0 (6.2-19)	<0.001
	Missing	71	10	
Hospital outcome	Survival	3799 (75.5%)	638 (79.5%)	0.017
	Death	1232 (24.5%)	165 (20.5%)	
**Hospital characteristics**				
Number of hospital beds category	<500	1123 (23.3%)	507 (63.8%)	<0.001
	500-1000	2519 (52.2%)	288 (36.2%)	
	>1000	1180 (24.5%)	0 (0%)	
	Missing	209	8	
ICU adjusted beds category^b^	<8	406 (8.1%)	307 (38.2%)	<0.001
	8-12	1879 (37.3%)	358 (44.6%)	
	>12	2746 (54.6%)	138 (17.2%)	
Teaching status of the hospital	Non-teaching	495 (9.8%)	306 (38.1%)	<0.001
	Teaching	4536 (90.2%)	497 (61.9%)	
Profit status of the hospital	For-profit	28 (0.6%)	84 (10.5%)	<0.001
	Non-profit	5003 (99.4%)	719 (89.5%)	
Possibility of extra beds inside ICU	No	3921 (77.9%)	696 (86.7%)	<0.001
	Yes	1110 (22.1%)	107 (13.3%)	
ICU nurse: patient ratio in daytime^c^	<0.5	1255 (24.9%)	0 (0%)	<0.001
	0.5-1	2382 (47.3%)	466 (58%)	
	>1	1394 (27.7%)	337 (42%)	

^a^IMCUs of any other hospital different from that of the ICU; ^b^number of ICU staffed beds adjusted for the ICUs having intermediate care beds inside considering two intermediate care beds inside ICU to be equivalent to one ICU bed; ^c^computed for only registered nurses. Data are number (N) with percentage or median with interquartile range (IQR). ICU: Intensive Care Unit; IMCU: Intermediate Care Unit (physically and administratively independent unit present in the hospital); LOC: Level of Care; SAPS: Simplified Acute Physiology Score.

There were 292 readmissions to ICUs with an IMCU and 40 readmissions to ICUs without an IMCU; five readmissions were excluded due to data inconsistencies. After the exclusion of readmissions with unknown hospital outcome, the hospital mortality after readmission was 37.7% (N = 103) and 27.0% (N = 10) in ICUs with and without an IMCU, respectively.

The variables entered into the multivariable analysis are reported in Table 2. The fully adjusted multivariable logistic regression analysis showed an OR of 0.63 (95% CI 0.45 to 0.88, *P* = 0.007) in favour of the presence of an IMCU. We performed a sensitivity analysis to check the robustness of this finding using SAPS 3, the SOFA and the NEMS scores instead of the SAPS II as acuity adjustor, by replacing SAPS II with each of these scores in the multivariate model. The OR with adjustment based on SAPS 3 was 0.66 (95% CI 0.46 to 0.94), 0.59 (95% CI 0.41 to 0.84) with adjustment based on SOFA, 0.55 (95% CI 0.39 to 0.78) with adjustment on NEMS. Severity of illness at ICU admission, presence of infection, hospital stay longer than seven days before ICU admission, and unplanned admission to the ICU were the patients’ factors significantly associated with an increased risk of hospital death, while ‘basic observation’ as the reason for ICU admission was a protective factor. Moreover, considering that Coronary Care Units are different from other IMCUs, we performed the multivariable analysis excluding the study patients admitted to the ICUs having a Coronary Care Unit as the only IMCU in the hospital. Only 31 (22.1%) of the 140 ICUs in a hospital with at least one independent IMCU had only one IMCU, and 12 of them were cardiac. The OR was 0.66 (95% CI 0.47 to 0.92, *P* = 0.015) in favour of the presence of an IMCU.

**Table 2:** **Multivariable model for the association with hospital mortality**

**Variable**		**OR**	**LL**	**UL**	***P*** **value**
IMCU in the hospital	No	1			
	Yes	0.63	0.45	0.88	0.007
‘Basic observation’ as ICU admission reason^a^	No	1			
	Yes	0.60	0.44	0.81	0.001
SAPS II	Per unit	1.07	1.06	1.08	<0.001
Gender	Female	1			
	Male	1.14	0.97	1.33	0.110
Infection	No	1			
	Yes	1.38	1.17	1.62	<0.001
Intra-hospital location before ICU admission	Emergency room	1			
	IMCU	1.09	0.76	1.56	0.635
	Other ICU	1.24	0.83	1.85	0.295
	Ward, other	1.16	0.93	1.45	0.200
Days in hospital before ICU admission	<24 h	1			
	1-7 days	1.13	0.92	1.40	0.249
	>7 days	1.79	1.35	2.36	<0.001
Adjusted number of ICU beds^b^	< 8	1			
	8-12	1.45	0.89	2.36	0.133
	>12	1.20	0.71	2.04	0.497
Type of admission to the ICU	Planned	1			
	Unplanned	1.42	1.11	1.83	0.006
Number of hospital beds	<500	1			
	500-1,000	2.29	1.61	3.25	<0.001
	>1,000	1.59	1.09	2.30	0.015
Possibility of allocating extra beds inside the ICU	No	1			
	Yes	0.98	0.70	1.37	0.905
ICU nurse: patient ratio in daytime	<0.5	1			
	0.5-1	1.16	0.79	1.71	0.449
	>1	1.30	0.80	2.10	0.285
Having intermediate care beds inside the ICU	No	1			
	Yes	0.99	0.73	1.35	0.968

^a^‘Basic observation’ generated according to the SOFA and NEMS variables for missing cases; ^b^number of ICU staffed beds adjusted for the ICUs having intermediate care beds inside considering two intermediate care beds inside ICU to be equivalent to one ICU bed. The presented odds ratios are adjusted on countries. OR: odds ratio, 95% confidence intervals reported as lower limit (LL) and upper limit (UL); LOC: Level of Care; *P* value: statistical significance. ICU: Intensive Care Unit; IMCU: Intermediate Care Unit: SAPS: Simplified Acute Physiology Score; SOFA: Sequential Organ Failure Assessment; NEMS: nursing workload index.

In a further sensitivity model, with an interaction term between presence of an IMCU and the reason for admission (‘basic observation’ versus other), the adjusted OR for the patients admitted to ICU for ‘basic observation’ was 1.15 (95% CI 0.65 to 2.03, *P* = 0.630) and that for patients requiring intensive treatment was 0.54 (95% CI 0.37 to 0.80, *P* = 0.002). The difference between these two ORs was statistically significant (*P* = 0.025). This suggests a possible interaction between the severity of illness of the patients with the effects of the presence or absence of an independent IMCU.

## Discussion

This prospective multinational European study is the first which demonstrates that adults admitted to ICUs of hospitals with an IMCU have significantly lower adjusted hospital mortality than those admitted to ICUs of hospitals without an IMCU. The adjusted IMCU effect in our study was close to one in the patients admitted to ICU for ‘basic observation’, and significantly lower than one (OR 0.54, 95% CI 0.37 to 0.80) for the patients admitted for other reasons, that is for those needing intensive treatment. Therefore, the finding of improved mortality associated with presence of an IMCU concerns the patients needing the intensive treatments performed in ICU.

We investigated only the effect of the presence of physically and administratively independent IMCUs on hospital mortality of ICU patients because intermediate care beds inside the ICU represent in many cases a management to match the level of care provided to ICU patients daily with the staff resources [35].

The large number of units and admissions collected is one of the major strengths of the present study. The quality of data collected is excellent as shown by the low number of missing data and patient exclusions, mostly due to being still in hospital at 90 days. The adjustment performed by the multivariable analysis has strongly moved the crude effect of a higher mortality for ICUs with an IMCU in an opposite direction. In non-randomised studies the case-mix adjustment is problematical but necessary [36]. In our study the adjustment was based on patient factors - including SAPS II, admission for ‘basic observation’, presence of infection, more than seven days in hospital before ICU admission and unplanned ICU admission. Besides the patients’ characteristics, we adjusted for countries because we suspected that mortality and health care management vary across countries. Additionally, some ICU and hospital characteristics have been introduced in the multivariate model to capture the hospital/ICU size (adjusted number of ICU beds, number of hospital beds). The organization of ICU was captured by the following factors: possibility of allocating extra beds inside the ICU, having intermediate care beds inside the ICU and ICU nurse to patient ratio during daytime hours. The size of the hospitals with and without an IMCU is different, being the former larger than the latter (median number of beds 665 vs. 294). A relationship between high volume and better outcome was reported in the EURICUS I database [37], for some high-risk surgical patients [38] and ICU cancer patients with septic shock [39], and a systematic review [40] confirmed this finding. Nevertheless, the volume-outcome relationship has been questioned [41] and a recent study found no correlation between standardized mortality ratio and ICU volume with only mechanically ventilated patients in very low-volume centres [42]. However, in our study we adjusted hospital mortality also for the size of the hospitals, which was strongly related to the volume of activity. Therefore, we have reason to believe that our finding is not due to the volume-outcome relationship.

Other relevant issues we had to deal with are the recently reported marked heterogeneity between European countries in the numbers of critical care beds [43], and the high number of ICUs from Central and Mediterranean countries present in our study. Fifteen of the seventeen countries participating in our study participated also in the European Surgical Outcomes Study (EuSOS) [44], which was designed to assess outcomes after non-cardiac surgery in Europe and collected data on 46,539 patients, 36,769 (79%) of which in the same countries as the present study. The weight of the geographic areas is different in EuSOS and in the present study, with Central and Western Europe prevalent in EuSOS, and Southern Europe and Mediterranean Countries prevalent in our study. When compared with the UK, the mortality rates recorded in EuSOS for three countries included also in the present study (Poland, Romania, and Ireland) are higher even after adjustment for the confounding variables identified in that study. Both this result [45-48] and the methodology [49,50] of EuSOS have been questioned, but an additional, more conservative, sensitivity analysis excluding 72 centres and 944 patients from the cohort remained consistent with the original conclusion that mortality was higher than expected, with significant variations between nations [51]. The methodology of our study is very different to EuSOS. However, we have taken into account the variations between countries and adjusted the IMCU effect on hospital mortality on countries.

In ICUs with an IMCU in the hospital, few patients (6.8%) were admitted from IMCU and less than one fifth of the survivors (18.8%) were transferred from ICU to IMCU. This percentage is not too different from that reported by Ranzani *et al*. who discharged 23% of their patients to IMCU [52]. Of note, the exclusion of the patients admitted to the 12 ICUs having a Coronary Care Unit as the only IMCU in the hospital did not change our results on hospital mortality. This finding may suggest that IMCUs, either cardiac or not, have an effect on hospital mortality of ICU patients, possibly because ICU-discharged patients having a late cardiac complication may benefit from these units.

There are several hypotheses that may explain how independent IMCUs can affect ICU patient outcome. First, the patients admitted to ICUs without an IMCU in the hospital could be less seriously ill than those admitted to ICUs with an IMCU as physicians may prefer an early, safer, transfer to ICU. Second, the patients admitted to ICUs without an IMCU in the hospital could be more seriously ill than those admitted to ICUs with an IMCU due to suboptimal care on ward, or deterioration not recognised in time. The first or the second hypothesis may prevail depending on the pressure on ICU beds. Our findings show that patients admitted to ICUs without an IMCU were less seriously ill than those admitted to ICUs with an IMCU in agreement with the first hypothesis. But the IMCU effect detected in the regression model cannot be explained by the severity of illness at admission as the model was adjusted for this confounding variable. Third, the patients admitted to ICUs without an IMCU in the hospital could have a longer ICU stay than those admitted to ICUs with an IMCU, needing more time to reach the level of nursing workload given in the ward. Fourth, the patients admitted to ICUs without an IMCU could be discharged from ICU too early, with a higher SOFA score and nursing workload, than those discharged from ICUs with an IMCU. In our study, the patient length of stay in ICUs without an IMCU was shorter than in ICUs with an IMCU. The SOFA and the NEMS scores at ICU discharge were similar in patients discharged from ICUs with and without an IMCU, suggesting the third and fourth hypotheses are wrong. We cannot exclude that things may be different at times of pressure on ICU beds but we do not have information about bed pressure.

The mechanisms explaining the lower in-hospital mortality in centres with an IMCU could be related to multiple different reasons. The monitoring and treatment provided by an IMCU to the patients needing it before ICU admission, and especially after ICU discharge, could have played a role, but cannot alone explain the main finding of the study. Possibly, the presence of an IMCU treating patients not admitted to ICU, especially in times of pressure on ICU beds, may have avoided an increase of the ICU staffing workload connected to the patient turnover (admissions, transfers and discharges). ICU staffing workload has been demonstrated to be associated with increased mortality [53], and West *et al*. [54] recently found a relationship between high staffing workload - measured by occupancy, admissions and transfers - and increased ICU mortality on 38,168 patients admitted to 65 UK ICUs collected in 1998. Therefore, we can hypothesise that an IMCU may have affected the in-hospital mortality of ICU patients also by a mechanism of reduction of ICU staffing workload. Unfortunately, our study did not assess the staffing workload of ICUs with and without IMCUs, and the functions of IMCUs where present, that is whether they facilitated earlier discharges of the ICU patients or ensured timely care for the patients deteriorating on the wards, or both.

The present study has some limitations. It is observational, because the decision to introduce an IMCU in hospitals or to assign patients to ICUs was outside the control of the investigators. It was performed only in ICUs participating on a voluntary basis, with some countries poorly represented, and hence participating ICUs did not necessarily represent the case mix of that country and our finding may not apply to all geographic locations. The selection of ICUs was not done randomly and can suffer from the effect of selection bias by the country coordinators. Moreover, the strict respect for patient anonymity did not give us solid clues to match each readmission with its first ICU admission. The effect of an IMCU was analysed only by the perspective of intensive care, thus nothing can be said about the possible effects of the presence, or absence, of IMCU on the outcome of patients hospitalized in other units. The small sample size and number of events in some participating centres is a limitation in our analysis because we modelled the mortality using methods for clustered data with centres as clusters. Nevertheless, our purpose was to assess the association between the presence of an IMCU and mortality, globally and not by centre, and the statistical power was sufficient since this association was statistically significant. Moreover, we cannot exclude that some confounding factors have been omitted in our model. Unfortunately, we did not assess characteristics and development of the teamwork in ICU, and whether the ICU and IMCU of the hospital shared the same staff. Teamwork is important to improve patient outcome [55], and the ICU and IMCU, when separated, should be prepared to join them for epidemics or mass casualties [56]. We could hypothesise a better patient outcome when the ICU and IMCU share the same staff compared to ICUs without an IMCU or with a totally independent IMCU, but we do not have data. Finally, we did not collect information about the daily ICU occupancy rate, or other measure of staffing workload which could indicate the ICU/IMCU relationship.

## Conclusions

This study is the first to provide evidence of the positive effect of having any physically and administratively independent intermediate care unit in the hospital on the mortality of adults admitted to ICU. This finding is relevant to health system and hospital managers who can find a scientific support to the decision to invest in having intermediate care beds in the hospital. Our study does not give evidence about the best staff to be involved in the management of intermediate care beds to improve patient outcome. Moreover, the differences in hospital and ICU beds, and characteristics of ICU-admitted patients found in the present study testify that settings with and without an IMCU may be basically different, and hence economic aspects may play a role in the decision of having intermediate care or not.

One of the main challenges now is to quantify and to compare the effects on patient outcomes and costs between two models: an independent IMCU operating in collaboration with ICUs and intermediate care beds inside large ICUs completely dependent from the ICU staff.

## Key messages

- IMCUs, which treat patients who require more care than could be provided on wards, may improve the outcome of ICU patients.
- We analyzed data collected on 5,834 patients admitted to 167 ICUs from 17 European countries.
- Patients admitted to ICUs with an IMCU in the hospital had a significantly reduced mortality, in comparison with patients admitted to ICUs without an IMCU in the hospital.

## Appendix

### Steering Committee

Maurizia Capuzzo, Ferrara, Italy (Lead Investigator)

Christophe Combescure, Geneva, Switzerland (Statistics)

Bertrand Guidet, Paris, France

Gaetano Iapichino, Milan, Italy

Rui Paulo Moreno, Lisboa, Portugal

Thomas Perneger, Geneva, Switzerland (Statistics)

Andrew Rhodes, London, United Kingdom

Andreas Valentin, Vienna, Austria

### Country Coordinators

*Austria*: Andreas Valentin; *Belgium*: Sandra Oeyen; *Czech Republic*: Martin Matejovic; *Denmark*: Palle Toft; *France*: Claude Martin; *Germany*: Hermann Wrigge; *Greece*: Despoina Koulenti; *Ireland*: Dorothy Breen; *Italy*: Rita Maria Melotti; *Norway*: Kristian Strand; *Poland*: Barbara Tamowicz; *Portugal*: Ricardo Matos; *Romania*: Natalia Hagau; *Spain*: Pedro Navarrete Navarro; *Turkey*: Yalim Dikmen; *United Kingdom*: Maurizio Cecconi.

### Study Unit Coordinators by country

#### Austria

Elisabeth Eggensperger (Hanusch Krankenhaus, Wien); Andreas Lerche (Krankenhaus Floridsdorf, Wien); Otmar Schindler (LKH Hoergas-Enzenbach, Gratwein); Lea Schirnhofer (University Hospital of Salzburg); Thomas Staudinger (General Hospital and Medical University of Vienna); Wolfgang Trebuch (LKH Wolfsberg); Andreas Valentin (Rudolfstiftung Hospital, Wien); Victoria Wieser (Otto Wagner Hospital, Wien).

#### Belgium

Vincent Collin (Cliniques de l’Europe - St Michel, Bruxelles); Pierre Damas, Paul Massion (CHU Sart Tilman, Liege); Nikolaas De Neve, Nathalie De Mey (Onze-Lieve-Vrouw Hospital, Aalst); Sandra Oeyen (Ghent University Hospital).

#### Czech Republic

Martin Matejovic (Charles University Hospital, Plzen); Igor Sas (University Hospital of Brno); Andrea Stoszkova (University Hospital, Hradec Kralove).

#### Denmark

Mads Konow Bøgebjerg (Sønderborg Sygehus); Mikkel Gybel (Glostrup Hospital); Malene Hollbaum Christiansen (AARHUS, Horsens); Jacob Kuhn, Torben F Jørgensen, Kristian Roerbaek Madsen, Marc Sørensen, Margit Vejen Stilling (Odense Universitets Hospital).

#### France

Jean-Michel Arnal (Hôpital Font-Pré, Toulon); Olivier Baldesi (Centre Hospitalier du Pays d’Aix, Aix en Province); Romain Barthélémy (Hôpital Lariboisiere, Paris); Pascal Beuret (Centre Hospitalier de Roanne); Gilles Blasco (CHU Besancon); Thierry Boulain (Hôpital de La Source, Centre Hospitalier Régional d’Orléans); Russell Chabanne (CHU de Clermont-Ferrand); Cédric Cleophax (Hôpital Lariboisiere, Paris); Francois Collet (Centre Hospitalier St Malo); Nadège Demars (CHU Antoine Béclère, Clamart); Mathieu Desmard (Groupe Hospitalier Universitaire Paris Nord Val de Seine); Michel Durand, Géraldine Dessertaine (Hôpital Michallon, Grenoble); Mathieu Egard (Centre Hospitalier de Mulhouse); Alexandre Faure (Hôpital Edouard Herriot, Lyon); Jean-Sebastien Faure, Russel Chabanne (CHU Gabriel Montpied, Clermont Ferrand); Jérôme Fichet (Hôpital Antoine Béclère, Clamart); Cathy Fleureau (Haut-Lévêque Hospital - CHU de Bordeaux); Gilles Francony (CHU de Grenoble); Marc Gainnier (CHU Timone, Marseille); Richard Galliot (Centre Hospitalier Felix Guyon, La Réunion); Stéphanie Houcke (Hôpital R. Salengro, Lille); Bernard Just (Centre Hospitalier Manchester, Charleville-Mezieres); Sigismond Lasocki (CHU Angers); Vincent Lassalle (Centre Hospitalier du Mans); Pierre Lavagne (CHU de Grenoble); Alain Leger (Hôpital Pellegrin, Bordeaux); Christine Lorut (Hotel Dieu, Paris); Mathieu Mattei (Centre Hospitalier de Brive); Herve Mentec (Centre Hospitalier Victor Dupouy, Argenteuil); Bertrand Meyssignac (Hôpital Nord, Marseille); Martine Nyunga (Centre Hospitalier de Roubaix); Vincent Peigne (Hôpital d’instruction des armées Percy, Clamart); François Philippart (Groupe hospitalier Paris Saint Joseph, Paris); Fabienne Plouvier (Hôpital Saint Esprit, Agen); Anne Renault, Solene Guinard (CHU de la Cavale Blanche, Brest); Antoine Roch (APHM - Hôpital Nord, Marseille); Hadrien Roze (Haut-Lévêque Hospital - CHU Bordeaux); Philippe Sarrabay (Haut-Lévêque Hospital - CHU Bordeaux); Carole Schwebel, Silvia Calvino Günther (CHU de Grenoble); Achille Sossou (Centre Hospitalier Emile Roux, Le Puy-en-Velay); Xavier Tchenio, Nicholas Sedillot (Hôpital Fleyriat, Bourg en Bresse); François Tinturier (CHU Amiens); Maurel Véronique (Hôpital Saint-Louis, Paris); Florent Wallet, Celine Bernet (Centre Hospitalier Lyon-Sud).

#### Germany

Justyna Swol (Berufsgenossenschaftliches Universitätsklinikum Bergmannsheil, Bochum); Hermann Wrigge, Philipp Simon (University Hospital Leipzig).

#### Greece

Elli Antypa (General Hospital ‘G. Gennimatas’, Thessaloniki); Theodoros Aslanidis, Aikaterini Euthimiou (General Hospital ‘A.H.E.P.A’, Thessaloniki); Dimitrios Babalis (General University Hospital of Larissa); Georgios Choutas, Vassiliki Tzani (251 Airforce General Hospital, Athens); Georgios Gkiokas, Dionysios Dellaportas (Aretaieion University Hospital, Athens); Maria Karapetsa (General University Hospital of Larissa); Vasilios Koulouras (University Hospital of Ioannina); Christina Kydona (Hippokration General Hospital, Thessaloniki); Magdalini Kyriakopoulou (Athens Chest Diseases Hospital Sotiria, Athens); George Kyriazopoulos (Lamia General Hospital); Athina Lavrentieva, Ioanna Kemanetzi (George Papanikolaou General Hospital, Thessaloniki); Efstratios Mainas (‘Hippokrateion’ General Hospital, Athens); Polychronis Malliotakis, Emmanouil Lilitsis (University General Hospital Heraklion); Pavlos Myrianthefs, Vassiliki Psalida (‘Agioi Anargyroi’ General Hospital, Athens); Spiridon Papanikolaou (Peiraiko Hospital, Athens); Anna Spring (Naval Hospital, Athens); Maria Stafylaraki (Sismanoglio General Hospital, Athens); Konstantinos Tasopoulos (IASO Thessalias Hospital, Larissa); Maria Theodorakopoulou, Despoina Koulenti (Attikon University Hospital, Athens); Pirros Tsakas (General Hospital, Arta).

#### Ireland

Joan Barry, Nicola Perry (Cork University Hospital); Janette Brohan, Dorothy Breen (Cork University Hospital); John Cahill, Ruth Aoibheann O’Leary (Mercy University Hospital, Cork); Erik Korba (Beaumont Hospital, Dublin); Roisin Nee, Mujtaba Ali (Limerick Regional Hospital); Nuala Treanor (South Infirmary Victoria University Hospital, Cork); Andrew Westbrook, Claire Nestor (St Vincent’s University Hospital, Dublin).

#### Italy

Maria Renata Bacchin, Aristide Morigi (Istituto Ortopedico Rizzoli, Bologna); Maria Barbagallo (Azienda Ospedaliero-Universitaria di Parma); Giuseppe Calicchio (Azienda Ospedaliera Universitaria S. Giovanni di Dio e Ruggi d’Aragona, Salerno); Cosetta Cantaroni (Ospedale Santa Maria della Scaletta, Imola); Paolo Chiarandini (Azienda Ospedaliero-Universitaria ‘S. Maria della Misericordia’, Udine); Nicola Cilloni (Ospedale Maggiore, Bologna); Andrea Neville Cracchiolo (Ospedale Civico di Cristina Benfratelli, Palermo); Massimo Girardis (AOU Modena); Paolo Lunghi (Ospedale Civile B. Ramazzini, Carpi, Modena); Giorgia Malewski (Ospedale M. Bufalini, Cesena); Demostene Marifoglou (Ospedale di Castel San Giovanni, Castel San Giovanni, Piacenza); Patrizia Murino (AORN Ospedale V. dei Colli-V. Monaldi, Napoli); Marco Adversi, Antonina Pigna (Azienda Ospedaliero-Universitaria S. Orsola-Malpighi, Bologna); Cristina Pinna, Francesco Ponzetta (Nuovo Ospedale S. Agostino Estense, Modena); Maurizio Postiglione (Ospedale S. Maria di Loreto, Napoli); Santi Maurizio Raineri (A.O.U. Policlinico ‘P. Giaccone’, Palermo); Paolo Spanu (Azienda Ospedaliera S. Paolo, Milano); Carlo Alberto Volta, Sofia Vitali (Azienda Ospedaliero-Universitaria S. Anna, Ferrara).

#### Norway

Finn H Andersen (Aalesund Hospital); Kristian Strand (Stavanger University Hospital).

#### Poland

Jerzy Drobiński (Hospital Ministry of Interior and Administration, Poznan); Anna Kanikowska (University Hospital of Lord’s Transfiguration, Poznan); Włodzimierz Kostyrka (Zespól Zakladów Opieki Zdrowotnej, Ostrów Wielkopolski); Mariusz Piechota (Uniwersytecki Szpital Kliniczny im WAM - Centralny Szpital Weteranów); Pawel Pietraszek (Szpital Wojewódzki w Zielonej Górze, Zielona Góra); Barbara Tamowicz (Regional Hospital, Poznan).

#### Portugal

Susana Afonso (Unidade de Cuidados Intensivos Neurocríticos, Hospital de São José, Centro Hospitalar de Lisboa Central); Sofia Beirão (Hospital Geral - Centro Hospitalar Universitário Coimbra); Luís Bento (Hospital de São José, Centro Hospitalar de Lisboa Central); Eduardo Almeida, Vânia Brito, Paula Mendes (Hospital Garcia de Orta, Almada); Ernestina Gomes (Unidade Local de Saude de Matosinhos); Elisabete Monteiro (Hospital São João, Porto); José Pedro Moura (Hospital Santa Luzia de Viana do Castelo); Teresa Oliveira (Hospital São João, Porto); Rosa Ribeiro (Hospital São Bernardo, Setubal); Zoryana Shumanska (Hospital de S. José, Centro Hospitalar de Lisboa Central); Rui Pedro Veiga (Hospital São João, Porto).

#### Romania

Calin Mitre (Institutul Regional de Gastroenterologie si Hepatologie, Cluj-Napoca); Constantin Bodolea (Municipal Hospital Cluj-Napoca); Serban Bubenek, Ioan Turconi (Institute of Emergency Cardiovascular Diseases ‘C.C. Iliescu’, Bucharest); Sanda Maria Copotoiu (University Emergency County Hospital Mures - SCJUMS, Targu Mures); Dan Corneci, Silvius Negoita (University Emergency Hospital Elias, Bucharest); Daniela Filipescu (Emergency Institute of Cardiovascular Disease, Bucharest); Ioana Grintescu, Irina Luca-Vasiliu (Clinical Emergency Hospital, Bucharest); Natalia Hagau, Dan Sebastian Dirzu (Clinical Emergency County Hospital of Cluj); Ciprian Hentia, Ovidiu Horea Bedreag (Timisoara Clinical Emergency County Hospital); Antonela Diana Muresan (‘N. Stancioiu’ Heart Institute, Cluj-Napoca); Adriana Stoicovici (Spitalul Judetean de Urgenta ‘Dr. Constantin Opris’, Baia Mare); Dana-Rodica Tomescu (Institutul Clinic Fundeni, Bucharest).

#### Spain

Luis Herrera Para, Laura Tarraga (Hospital Universitario Santa Lucia de Cartagena); Maria Cruz Martin Delgado, Nuria Camino Redondo (Hospital de Torrejon, Madrid); Pablo Monedero, Cisse Mbongo (Clinica Universidad de Navarra, Pamplona); Amaia Quintano (Hospital Universitario de Álava - Santiago, Vitoria-Gazteiz).

#### Switzerland

Hervé Zender (Hôpital Neuchâtelois, La Chaux-de-Fonds).

#### Turkey

Nesrin Alpaslan (Bayindir Hastanesi Icerenkoy, Istanbul); Yalim Dikmen (Cerrahpasa Medical School Hospital, Istanbul); Derya Gokcinar (Ankara Numune Teaching and Research Hospital, Ankara); Mustafa Gonullu (Tepecik Egitim ve Arastirma Hastanesi, Izsmir); Zehra Hatípoğlu (Çukurova University Faculty of Medicine, Adana); Namik Ozcan (Ankara Training and Research Hospital, Ankara); Yasemin Tekdőş (Bakirkoy Dr Sadi Konuk Researching and Education Hospital, Istanbul).

#### United Kingdom

Casiano Barrera-Groba, Laura Ortiz-Ruiz de Gordoa (Royal Sussex County Hospital, Brighton); Maurizio Cecconi, Claudia Ebm (St. George’s Healthcare Trust, London); Aylwin Chick, Alice Tipton (Northern General Hospital, Sheffield); Julius Cranshaw (Royal Bournemouth Hospital); Paul Downie (Gloucestershire Hospitals NHS Foundation Trust); Tariq Husain (Northwick Park Hospital, London); Bhushan Joshi, Rakesh Vaja (Glenfield Hospital, Leicester); Martin Kendra (Heatherwood and Wexham Park Hospital NHS Trust, Slough); Marlies Ostermann, Victoria Stables, Christina Wlodek, Louise McWhirter (Guy’s Hospital, London); Marlies Ostermann, Thomas Wilson, Anna Williams, Priya Sriskandarajah (St Thomas Hospital, London); Tamas Szakmany (Royal Glamorgan Hospital, Llantrisant); Malcolm Watters (Great Western Hospital, Swindon); Mehrun Zulieka, Peter Carvalho (Royal Surrey County, Guilford).

## Additional files

Additional file 1
**Quality control on 281 (4%) of the 6,401 admissions to 169 ICUs.** Correlation coefficient reported as kappa or intraclass correlation. Additional file 2
**Number of study ICUs and patient admissions, and presence of Intermediate Care Units in the hospital per country.**
Additional file 3
**Organisational characteristics of the study ICUs, with and without Intermediate Care Unit in the hospital.**
Additional file 4
**Reasons for patient admission to ICUs with and without Intermediate Care Unit in the hospital.**

## Abbreviations

CCC: Coordination and Communications Centre
GEE: generalized estimating equation
ICU: Intensive Care Unit
IMCU: Intermediate Care Unit
LOC: Level of Care
NEMS: nine equivalents of nursing manpower use score (nursing workload index)
SAPS: simplified acute physiology score
SOFA: sequential organ failure assessment
