# Supplementary material for: Hospital mortality of adults admitted to Intensive Care Units in hospitals with and without Intermediate Care Units: a multicentre European cohort study
Source: Crit Care. 2014 Oct 9;18(5):551. doi: 10.1186/s13054-014-0551-8 (PMC4261690; doi:10.1186/s13054-014-0551-8)
Supplement: Additional file 1 — Quality control on 281 (4%) of the 6,401 admissions to 169 ICUs. Correlation coefficient reported as kappa or intraclass correlation. [file 13054_2014_551_MOESM1_ESM.doc]

**Hospital mortality of adults admitted to Intensive Care Unit in hospitals with and without Intermediate Care Unit: A multicentre European cohort study**

Maurizia Capuzzo, Carlo Alberto Volta, Tania Tassinati, Rui Paulo Moreno, Andreas Valentin, Bertrand Guidet, Gaetano Iapichino, Claude Martin, Thomas Perneger, Christophe Combescure, Antoine Poncet, Andrew Rhodes on behalf of the Working Group on Health Economics of the European Society of Intensive Care Medicine

**Additional file 1**: Quality control on 281 (4%) of the 6401 admissions to 169 ICUs.

**Coefficient Kappa Intraclass correlation**

Gender 0.955

Age 0.992

Hospital admission date 0.939

Hospital admission hour 0.906

Transfer to higher LOC before ICU admission 0.842

ICU admission date 0.807

ICU admission hour 0.991

ICU Readmission 0.838

Planned/unplanned ICU admission 0.933

Intra-hospital location before ICU admission 0.965

Chronic diseases 0.972

Admission medical, emergent or elective surgical 0.988

SAPS II score 0.985

SAPS 3 BOX I 0.980

SAPS 3 BOX II 0.972

SAPS 3 BOX III 0.981

ICU discharge date 0.996

ICU discharge hour 0.937

Vital status at ICU discharge 0.979

Hospital discharge date 1

Hospital discharge hour 0.919

Vital status at hospital discharge 0.973

Transfer to higher LOC after ICU discharge 0.853
