# Supplementary material for: Hospital mortality of adults admitted to Intensive Care Units in hospitals with and without Intermediate Care Units: a multicentre European cohort study
Source: Crit Care. 2014 Oct 9;18(5):551. doi: 10.1186/s13054-014-0551-8 (PMC4261690; doi:10.1186/s13054-014-0551-8)
Supplement: Additional file 2 — Number of study ICUs and patient admissions, and presence of Intermediate Care Units in the hospital per country. [file 13054_2014_551_MOESM2_ESM.doc]

**Hospital mortality of adults admitted to Intensive Care Unit in hospitals with and without Intermediate Care Unit:**

**A multicentre European cohort study**

Maurizia Capuzzo, Carlo Alberto Volta, Tania Tassinati, Rui Paulo Moreno, Andreas Valentin, Bertrand Guidet, Gaetano Iapichino, Claude Martin, Thomas Perneger, Christophe Combescure, Antoine Poncet, Andrew Rhodes on behalf of the Working Group on Health Economics of the European Society of Intensive Care Medicine

**Additional file 2**: Number of study ICUs and admissions, and presence of Intermediate Care Unit in the hospital per country

| **Country** | **ICUs** |  | **Admissions** | **N. admissions per ICU** | | **ICUs with** |  | **ICUs without** |  |
| --- | --- | --- | --- | --- | --- | --- | --- | --- | --- |
|  | **N** | **%** | **N** | **Median** | **IQR** | **IMCU** | **%** | **IMCU** | **%** |
|  |  |  |  |  |  |  |  |  |  |
| Austria | 7 | 4.2 | 151 | 23 | 15 - 25 | 6 | 85.7 | 1 | 14.3 |
| Belgium | 4 | 2.4 | 278 | 68 | 61 - 77 | 3 | 75.0 | 1 | 25.0 |
| Czech Republic | 3 | 1.8 | 108 | 35 | 31 - 41 | 3 | 100.0 | 0 |  |
| Denmark | 6 | 3.6 | 275 | 37 | 26 - 58 | 3 | 50.0 | 3 | 50.0 |
| France | 43 | 25.7 | 1684 | 36 | 27 - 51 | 42 | 97.7 | 1 | 2.3 |
| Germany | 2 | 1.2 | 156 | 78 | 67 - 89 | 2 | 100.0 | 0 |  |
| Greece | 20 | 12.0 | 386 | 18 | 13 - 21 | 14 | 70.0 | 6 | 30.0 |
| Ireland | 8 | 4.8 | 257 | 30 | 27 - 40 | 4 | 50.0 | 4 | 50.0 |
| Italy | 18 | 10.8 | 557 | 26 | 20 - 39 | 15 | 83.3 | 3 | 16.7 |
| Norway | 2 | 1.2 | 47 | 24 | 19 - 28 | 1 | 50.0 | 1 | 50.0 |
| Poland | 6 | 3.6 | 126 | 20 | 17 - 23 | 4 | 66.7 | 2 | 33.3 |
| Portugal | 11 | 6.6 | 285 | 25 | 22 - 33 | 11 | 100.0 | 0 |  |
| Romania | 12 | 7.2 | 770 | 74 | 51 - 76 | 11 | 91.7 | 1 | 8.3 |
| Spain | 4 | 2.4 | 283 | 75 | 53 - 93 | 3 | 75.0 | 1 | 25.0 |
| Switzerland | 1 | 0.6 | 68 |  |  | 0 |  | 1 | 100.0 |
| Turkey | 7 | 4.2 | 147 | 21 | 12-27 | 7 | 100.0 | 0 |  |
| UK | 13 | 7.8 | 823 | 67 | 37 - 85 | 11 | 84.6 | 2 | 15.4 |
| Total | 167 | 100.0 | 6,401 | 32 | 20 - 53 | 140 | 83.8 | 27 | 16.2 |
