# Supplementary material for: Hospital mortality of adults admitted to Intensive Care Units in hospitals with and without Intermediate Care Units: a multicentre European cohort study
Source: Crit Care. 2014 Oct 9;18(5):551. doi: 10.1186/s13054-014-0551-8 (PMC4261690; doi:10.1186/s13054-014-0551-8)
Supplement: Additional file 3 — Organisational characteristics of the study ICUs, with and without Intermediate Care Unit in the hospital. [file 13054_2014_551_MOESM3_ESM.doc]

**Hospital mortality of adults admitted to Intensive Care Unit in hospitals with and without Intermediate Care Unit: A multicentre European cohort study**

Maurizia Capuzzo, Carlo Alberto Volta, Tania Tassinati, Rui Paulo Moreno, Andreas Valentin, Bertrand Guidet, Gaetano Iapichino, Claude Martin, Thomas Perneger, Christophe Combescure, Antoine Poncet, Andrew Rhodes on behalf of the Working Group on Health Economics of the European Society of Intensive Care Medicine

**Additional file 3**: Organisational characteristics of the study ICUs

| **ICUs** |  | **with** | **%** | **without** | **%** | **p *** |
| --- | --- | --- | --- | --- | --- | --- |
|  |  | **IMCU** |  | **IMCU** |  |  |
| N. of ICUs |  | 140 | 84 | 27 | 16 |  |
| Number of acute hospital beds | <500 | 40 | 29 | 19 | 70 | <0.001 |
|  | 500-1000 | 61 | 44 | 7 | 26 |  |
|  | >1000 | 32 | 23 | 0 | 0 |  |
|  | missing | 7 |  | 1 |  |  |
| Teaching status of the hospital | no | 122 | 23 | 19 | 30 | 0.056 |
|  | yes | 18 | 87 | 8 | 70 |  |
| Profit status of the hospital | non-profit | 134 | 96 | 24 | 89 | 0.331 |
|  | for profit | 6 | 4 | 3 | 11 |  |
| Number of ICU staffed beds | <8 | 19 | 14 | 11 | 41 | <0.001 |
|  | 8-12 | 57 | 41 | 13 | 48 |  |
|  | >12 | 64 | 46 | 3 | 11 |  |
| Adjusted N of ICU staffed beds ^a^ | <8 | 21 | 15 | 13 | 48 | <0.001 |
|  | 8-12 | 62 | 44 | 12 | 44 |  |
|  | >12 | 57 | 41 | 2 | 7 |  |
| Intermediate care beds inside the ICU | no | 89 | 64 | 20 | 74 | 0.407 |
|  | yes | 51 | 36 | 7 | 26 |  |
| Possibility of allocating extra beds inside | no | 113 | 81 | 22 | 81 | 0.862 |
|  | yes | 27 | 19 | 5 | 19 |  |
| Physician/Patient ratio in daytime ^b^ | <0.2 | 7 | 5 | 2 | 7 | 0.603 |
|  | 0.2-0.5 | 78 | 56 | 17 | 63 |  |
|  | >0.5 | 55 | 39 | 8 | 30 |  |
| Physician/Patient ratio in night time ^b^ | <0.2 | 64 | 46 | 15 | 56 | 0.585 |
|  | 0.2-0.5 | 66 | 47 | 11 | 41 |  |
|  | >0.5 | 10 | 7 | 1 | 4 |  |
| Nurse/Patient ratio in daytime ^c^ | <0.5 | 17 | 12 | 3 | 11 | 0.543 |
|  | 0.5-1 | 81 | 58 | 13 | 48 |  |
|  | >1 | 42 | 30 | 11 | 41 |  |
| Nurse/Patient ratio in night time ^c^ | <0.5 | 43 | 31 | 9 | 33 | 0.455 |
|  | 0.5-1 | 73 | 52 | 11 | 41 |  |
|  | >1 | 24 | 17 | 7 | 26 |  |

* p: statistical significance (Chi-square test)

^a^ Number of ICU staffed beds adjusted for the ICUs having intermediate care beds inside considering 2 intermediate care beds inside ICU to be equivalent 1 ICU bed

^b^ Physicians include residents, and the ratio was computed according to the ICU staffed beds adjusted for the ICUs having intermediate care beds

^c^ Nurses include registered nurses and nurse aids, and the ratio was computed according to the ICU staffed beds adjusted for the ICUs having intermediate care beds
