# Supplementary material for: Hospital mortality of adults admitted to Intensive Care Units in hospitals with and without Intermediate Care Units: a multicentre European cohort study
Source: Crit Care. 2014 Oct 9;18(5):551. doi: 10.1186/s13054-014-0551-8 (PMC4261690; doi:10.1186/s13054-014-0551-8)
Supplement: Additional file 4 — Reasons for patient admission to ICUs with and without Intermediate Care Unit in the hospital. [file 13054_2014_551_MOESM4_ESM.doc]

**Hospital mortality of adults admitted to Intensive Care Unit in hospitals with and without Intermediate Care Unit: A multicentre European cohort study**

Maurizia Capuzzo, Carlo Alberto Volta, Tania Tassinati, Rui Paulo Moreno, Andreas Valentin, Bertrand Guidet, Gaetano Iapichino, Claude Martin, Thomas Perneger, Christophe Combescure, Antoine Poncet, Andrew Rhodes on behalf of the Working Group on Health Economics of the European Society of Intensive Care Medicine

**Additional file 4**: Reasons for patient admission to ICUs with and without InterMediate Care Unit (IMCU) in the hospital. DIC: Disseminated Intravascular Coagulation; CPD: chronic pulmonary disease; ALI: Acute Lung Injury; ARDS: Adult Respiratory Distress Syndrome.

| **ICUs** |  | **with** | **%** | **without** | **%** | **p *** |
| --- | --- | --- | --- | --- | --- | --- |
|  |  | **IMCU** |  | **IMCU** |  |  |
| N of patients |  | 5031 |  | 803 |  |  |
| Missing information^a^ |  | 37 | 1 | 6 | 1 |  |
| Basic observational^b^ | no | 3804 | 76 | 475 | 59 | <0.001 |
|  | yes | 1227 | 24 | 328 | 41 |  |
| Cardiovascular | anaphylactic, mixed and undefined shock | 25 | 0 | 2 | 0 | <0.001 |
|  | cardiac arrest | 258 | 5 | 28 | 3 |  |
|  | cardiac failure without shock | 96 | 2 | 6 | 1 |  |
|  | cardiogenic shock | 115 | 2 | 16 | 2 |  |
|  | chest pain (with ECG changes) | 56 | 1 | 16 | 2 |  |
|  | hypertensive crisis | 15 | 0 | 3 | 0 |  |
|  | hypovolemic shock | 106 | 2 | 18 | 2 |  |
|  | other cardiovascular reason | 178 | 4 | 57 | 7 |  |
|  | rhythm disturbances | 64 | 1 | 19 | 2 |  |
|  | septic shock | 308 | 6 | 23 | 3 |  |
| Digestive | acute abdomen | 175 | 3 | 29 | 4 | 0.253 |
|  | bleeding | 121 | 2 | 23 | 3 |  |
|  | other digestive reason | 176 | 3 | 18 | 2 |  |
|  | severe pancreatitis | 42 | 1 | 5 | 1 |  |
| Haematological | haemorrhagic syndrome (including DIC) | 39 | 1 | 3 | 0 | 0.459 |
|  | other haematological reason | 35 | 1 | 4 | 0 |  |
|  | severe haemolysis | 4 | 0 | 1 | 0 |  |
| Hepatic | liver failure | 36 | 1 | 5 | 1 | 1 |
|  | other hepatic reason | 29 | 1 | 4 | 0 |  |
| Metabolic | acid-base and/or electrolyte disturbance | 141 | 3 | 11 | 1 | 0.073 |
|  | hypo and hyperglycemias | 13 | 0 | 4 | 0 |  |
|  | hypo and hyperthermia | 23 | 0 | 1 | 0 |  |
|  | other metabolic reason | 17 | 0 | 3 | 0 |  |
| Neurological | coma, stupor, obtunded ^c^ | 459 | 9 | 33 | 4 | 0.003 |
|  | focal neurological deficit | 37 | 1 | 12 | 1 |  |
|  | intracranial mass effect | 123 | 2 | 11 | 1 |  |
|  | other neurological reason | 123 | 2 | 14 | 2 |  |
|  | seizures | 85 | 2 | 11 | 1 |  |
| Renal | acute obstructive renal failure | 17 | 0 | 3 | 0 | 0.029 |
|  | Acute kidney failure | 60 | 1 | 6 | 1 |  |
|  | acute pre-renal failure | 64 | 1 | 3 | 0 |  |
|  | other renal reason | 56 | 1 | 14 | 2 |  |
| Respiratory | acute respiratory failure on CPD | 362 | 7 | 44 | 5 | <0.001 |
|  | ALI and ARDS | 254 | 5 | 9 | 1 |  |
|  | other respiratory reason | 391 | 8 | 88 | 11 |  |
| Other | severe trauma | 240 | 5 | 32 | 4 | 0.798 |
|  | other | 255 | 5 | 37 | 5 |  |

^*^ p value: statistical significance according to Fisher exact test

^a^ information about reason for ICU admission was considered as missing in the lack of any reason for ICU admission and if basic observational was either missing or “no”

^b^ "Basic observational" category generated according to the SOFA and NEMS variables for missing cases

^c^ including also vigilance disturbances, confusion, delirium
